# Supplementary material for: Improvement of glymphatic–lymphatic drainage of beta-amyloid by focused ultrasound in Alzheimer’s disease model
Source: Sci Rep. 2020 Sep 30;10:16144. doi: 10.1038/s41598-020-73151-8 (PMC7527457; doi:10.1038/s41598-020-73151-8)
Supplement: Supplementary file 1 — Supplementary Figures. [file 41598_2020_73151_MOESM1_ESM.docx]

**Improvement of glymphatic-lymphatic drainage of beta-amyloid by focused ultrasound in Alzheimer’s disease model**

Youngsun Lee^1,2†^, Yoori Choi ^1†*^, Eun-Joo Park^3,4†*^, Seokjun Kwon^1,2^, Hyun Kim^1,2^, Jae Young Lee^4^, Dong Soo Lee^1,2,*^

**Supplementary data**

**
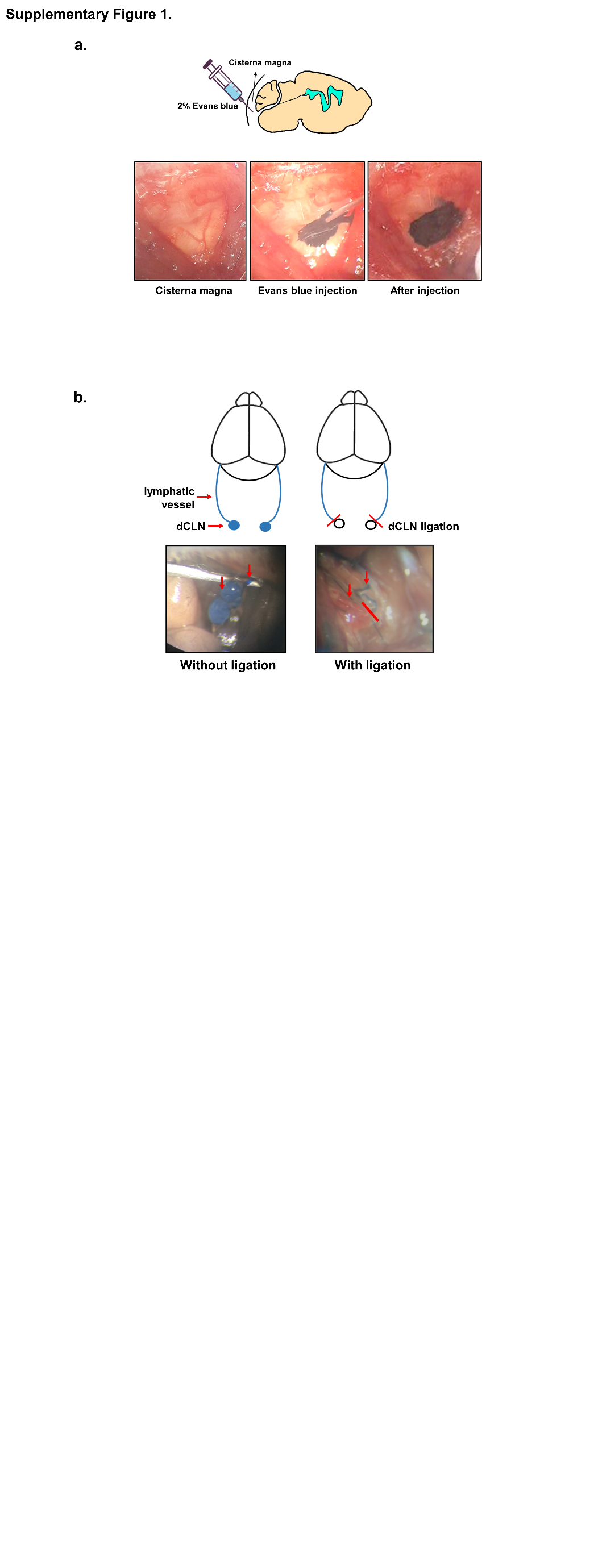
**

**Supplementary Figure 1. Validation of dCLN ligated animal model**

**a**. Evans blue was injected into cisterna magna of mice to confirm the ligation of lymphatics. **b**. At 30 minutes after Evans blue injection into cisterna magna, dCLN changed to blue. On the other hand, dCLN did not turn blue after dCLN ligation. Red arrows indicate lymphatic vessel and dCLN. And the red line represents dCLN ligation.

**
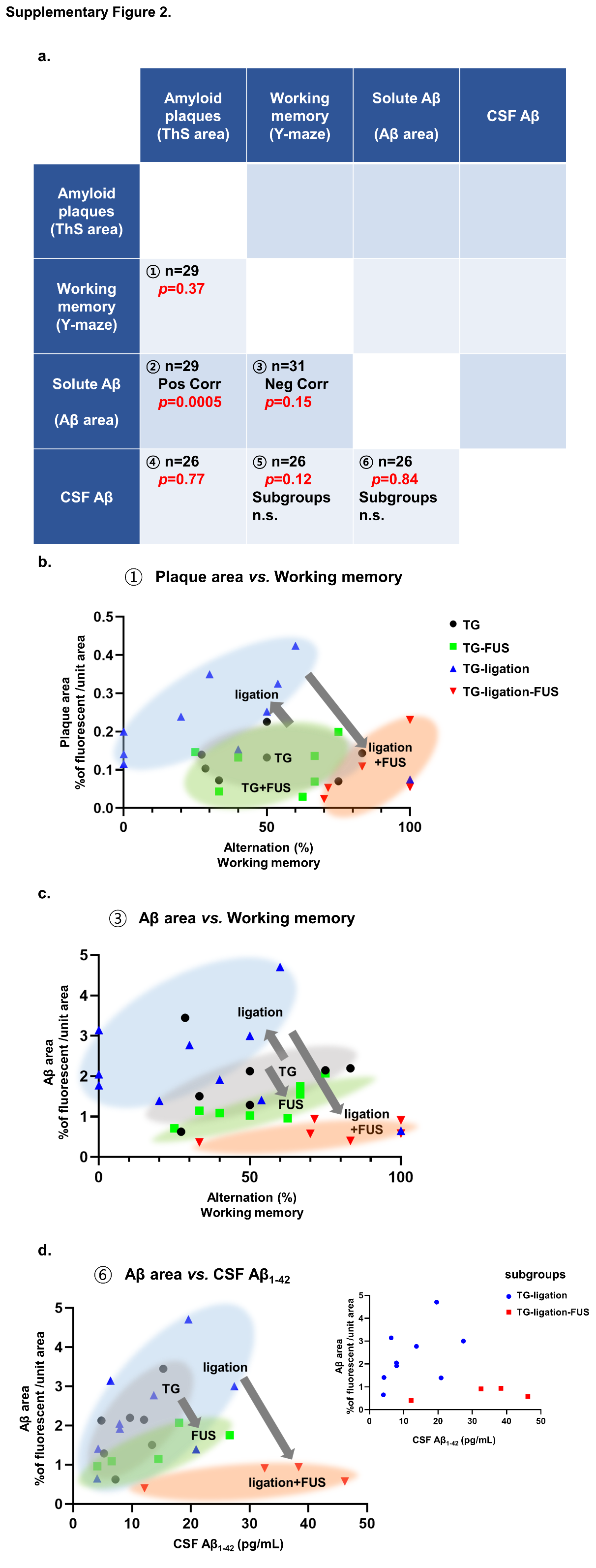
**

**
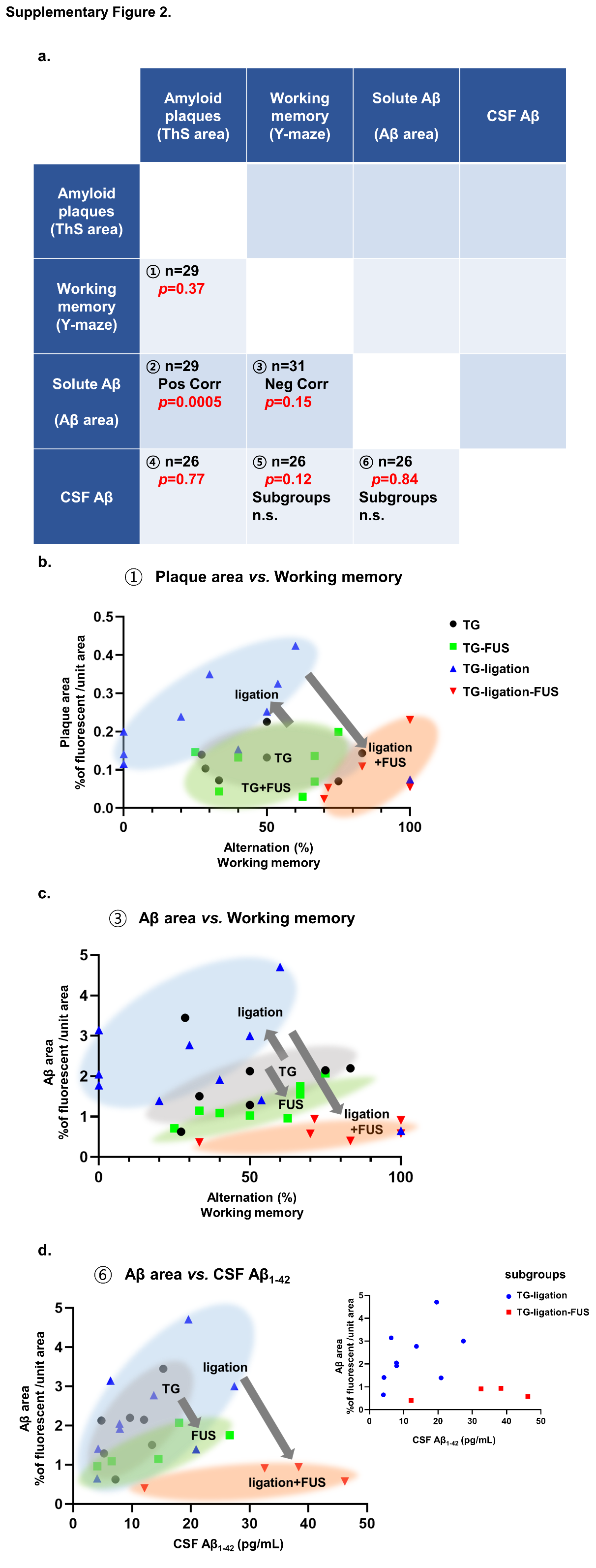
**

**Supplementary Figure 2. Correlation between measured values from individuals**

**a**. The correlation table between measured values from individuals summarized the number of individuals and their *p*-values. *P* values were obtained from simple linear regression between the values (Prism software). Pos Corr and Neg Corr mean positive and negative correlation, respectively. Subgroups include dCLN ligated mice and dCLN ligated mice with FUS-MB. And n.s. means not significant. **b**. Correlation graph between plaque area and working memory (Y-maze) in all individuals. **c**. Correlation graph between Aβ area and working memory in all individuals. **d**. Correlation graph between Aβ area and CSF Aβ_1-42_ in all individuals. Ellipses represent the distribution of individuals in each group. Gray arrows indicate the direction of changes in the group distribution depending on the conditions.
